# Developing cerium modified gold nanoclusters for the treatment of advanced-stage rheumatoid arthritis

**DOI:** 10.1016/j.mtbio.2022.100331

**Published:** 2022-06-23

**Authors:** Sen Lin, Wei Gao, Jiachen Sun, Kai Gao, Dan Li, Xifan Mei

**Affiliations:** a Department of Orthopedics, Third Affiliated Hospital of Jinzhou Medical University, Jinzhou, PR China; b Key Laboratory of Medical Tissue Engineering, Jinzhou Medical University, Jinzhou, PR China; c Department of Rheumatology and Immunology, First Affiliated Hospital of Jinzhou Medical University, Jinzhou, PR China; d Department of Orthopedics, Jining No. 1 People's Hospital, Jining, PR China; e College of Pharmacy, Jinzhou Medical University, Jinzhou, PR China

**Keywords:** Immunochemotherapy, RA classification, Advanced-stage rheumatoid arthritis, Gold nanoclusters

## Abstract

Rheumatoid arthritis (RA) is an autoimmune-mediated inflammatory disease that seriously threatens patients’ life. Different stages of RA require different treatments, but the accurate classification of the RA remains challenging. Herein, we conducted an in-depth study of 73 RA patients to investigate RA development. CD 19, a biomarker of B cell dysfunction, was found to be strongly associated with the development of severe symptoms. On the other hand, CD19 was significantly reduced, when effective clinical treatment relieved the symptoms. Therefore, it is proposed that B cell-inducing factors are important for the development of RA to the advanced stage and can be used to assist in the accurate classification of RA development. Furthermore, we speculated that drugs that could properly modulate B cells might have efficacy in advanced-stage RA. From this perspective, R-dihydrolipoic acid (R-DHLA)-stabilized cerium-modified gold nanoclusters (AuNCs) (R-DHLA-AuNCs-Ce) (∼3.4 ​nm) were developed for comprehensive treatment of advanced-stage RA. According to our established rat models of collagen-induced arthritis (CIA), R-DHLA-AuNCs-Ce restored the comprehensive changes of cytokines to a normal state by regulating B cell activity within 24 ​h. Furthermore, the immune responses elicited by B cells were memory-suppressed after detachment from R-DHLA-AuNCs-Ce and the advanced symptoms of RA in CIA rats were successfully reversed to a healthy state. Compared to clinical drugs such as methotrexate (MTX) and etanercept, R-DHLA-AuNCs-Ce were found to more efficiently suppress B cell immunity mitigating advanced-stage RA.

## 1 Introduction

Rheumatoid arthritis (RA) is a chronic inflammatory disease that characterizes by persistent synovitis, and cartilage destruction, and leads to disability and death [1,2]. The symptoms in advanced-stage RA patients are more complicated and require challenging treatment that is different from the early-stage RA [1,2]. Most of the current RA classifications are based on the severity of symptoms for humans, but it is difficult to accurately classify RA development based on the non-invasive diagnosis. Some anti-inflammatory drugs can suppress inflammation to a certain extent and improve RA symptoms to a lesser extent [[3], [4], [5]]. However, after long-term medication, the continuous improvement effect is not obvious, and it is likely to produce a variety of side effects. Given the pain caused by RA to human beings, it is very urgent to find a simple way to evaluate the development of RA and find corresponding treatment methods.

Immunotherapy refers to the treatment of diseases by inducing, enhancing, or suppressing the immune response [6,7]. A common strategy is to regulate immune cells in the early stage and fight the disease through the action of immune cells in the later stage, thereby reducing drug dependence [8]. This strategy has been largely investigated for the treatment of cancers, but the research on other diseases is limited. B cells are important immune cells that not only produce antibodies but also act as potent antigen-presenting cells. Clinical studies have been performed for B cell-based cancer vaccines [9,10]. This indicates that B cell-based immunotherapy holds promise for fighting against subsequent disease-causing factors. Immune memory is a unique property of B cells. It can “store” stimulus information and produce an equally effective response when a similar stimulus is encountered again. One of the most important symptoms of RA is chronic inflammation. From B-cell-based cancer immunotherapy, we reasoned that if B cells could be stimulated by a drug and then isolated from that drug to suppress inflammatory factors based on immunological memory, this strategy might have great promise for the treatment of advanced-stage RA without long-term medication.

**Scheme 1:**
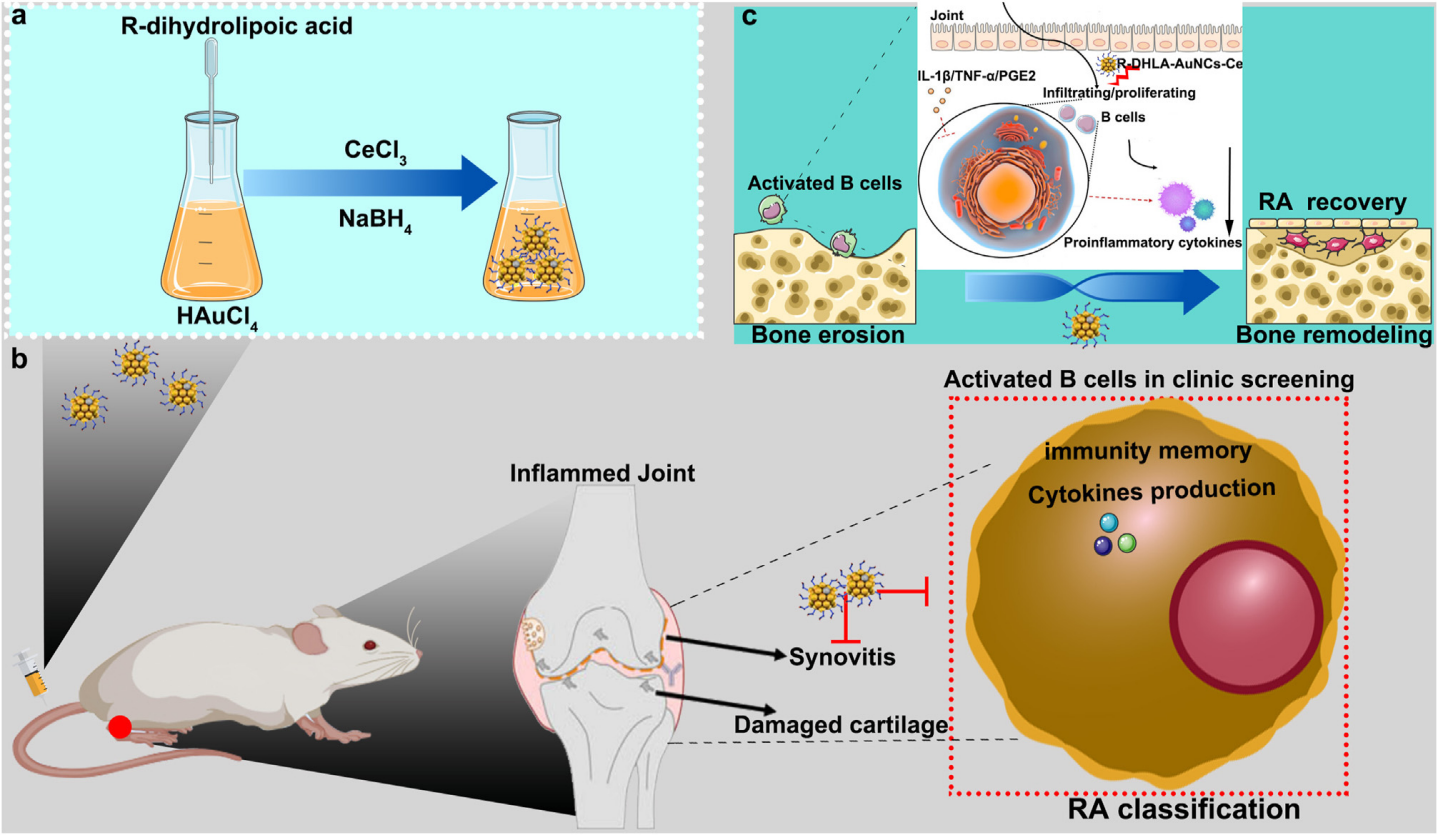
(a) One-pot synthesis of R-DHLA-AuNCs-Ce; (b) B cell immune responses were used to assess RA classification. The immune memory effect of B cells is stimulated by R-DHLA-AuNCs-Ce, which is used to continuously treat advanced-stage RA and reduce drug dependence. (c) The efficacy of the strategy includes anti-inflammatory, promoting osteogenesis, inhibiting osteoclast, joint repair, etc.

Nanomedicines have various advantages such as decreased adverse events, enhanced biological abilities, and improved biosafety [11,12], as compared to traditional drugs. For instance, cerium oxide-based nanoparticles have been used for chemotherapy [13]; On the other hand, the gold nanoclusters (AuNCs) showed immunotherapy effects for cancer treatment [14,15]. Given the performance of AuNCs and Ce-based nanomaterials in immunotherapy and chemotherapy, we expect that combining Ce with AuNCs may achieve a synergistic effect on B cells. Herein, we developed R-dihydrolipoic acid (R-DHLA) stabilized cerium-modified gold nanoclusters (AuNCs) (R-DHLA-AuNCs-Ce) and investigated their therapeutic effects compared to clinical drugs for relieving RA symptoms (Scheme 1). R-DHLA-AuNCs-Ce was first obtained by a one-pot synthesis method with simplifying mixing of R-DHLA, HAuCl_4,_ and CeCl_3_. After adding the reducing agents (NaBH_4_), R-DHLA-AuNCs-Ce were obtained. Unlike many nanomedicines for the treatment of RA that needed complicated modifications or functionalizations [[16], [17], [18]], the product of R-DHLA-AuNCs-Ce was purified by dialysis and could be directly used as a drug for the treatment of advanced-stage RA. The entire sample synthesis, purification, and use do not require any organic solvents or harmful reagents. Furthermore, we found B cell-inducing factors could be evaluated to accurately classify the RA stages based on clinical investigations. And R-DHLA-AuNCs-Ce just successfully regulated B-cell balance, which maintained the inflammatory factors such as tumor necrosis factor α (TNF-α), interleukin 6 (IL-6), and IL-1β to normal levels in vitro and in vivo. Additionally, the runt-related transcription factor 2 (Runx2), bone morphogenetic protein 2 (BMP-2), osteocalcin (OCN), and osteopontin (OPN) levels were found to be promoted in MC3T3-E1 cell culture under inflammatory conditions by the stimulation of R-DHLA-AuNCs-Ce, indicating the promotion of osteogenesis. At the same time, the osteoclasts that might induce bone loss under pathologic conditions were inhibited. After stopping the medication after 28 ​h, we observed persistent symptom relief in a CIA rat model of advanced RA, with eventual joint recovery. This indicates that the immune system memorized the stimulation of B cells by R-DHLA-AuNCs-Ce and continued to exert therapeutic effects. Compared with clinical RA drugs including methotrexate (MTX) and etanercept, R-DHLA-AuNCs-Ce has the following advantages: 1) The inflammation was suppressed more remarkably, which enabled both synovium and bone to regenerate more efficiently; 2) The more pronounced improvement in RA symptoms at a faster time greatly reduces the duration of medication. 3) The systemic toxicity and side effects were also reduced due to the efficient clearance. Overall, we successfully used B cell-inducing factors to find a way to classify the development of RA and constructed a rat CIA model of advanced stage RA. We use R-DHLA-AuNCs-Ce to stimulate B cells and suppress the related immune response in the early stage. Though R-DHLA-AuNCs-Ce was not used in the later stage, the immune memory of B cells continued to treat the symptoms of advanced-stage RA. This work not only opens up new directions for the effective assessment and treatment of this incurable advanced-stage RA but also contributes to reducing the long-term use of anti-inflammatory drugs.

## 2 Materials and methods

### 2.1 Chemical reagents and instrument

Anti-TNF-α antibody and prostaglandin E2 (PGE2) ELISA kit were obtained from Abcam. IL-1β and TNF-α ELISA kits were obtained from Absin. EDTA-Decalcifying-fluid was obtained from Boster. Freund's adjuvant was obtained from Sigma. Collagen type II, hematoxylin & eosin staining kit and Masson's trichrome stain kits were purchased from Solarbio (TEM) and high-resolution transmission electron microscopy (HR-TEM) images were recorded using Tecnai™ G2 F30 Series. Energy-dispersive X-ray spectroscopy (EDS) was performed using JEM 2100F. Inductively coupled plasma mass spectrometry (ICP-MS) was used to determine the concentration of gold and cerium in the cluster drugs.

### 2.2 Synthesis of the cluster drugs

R-DHLA-AuNCs-Ce were synthesized according to our previous work with modifications [19]: 5.2 ​mg of R-lipoic acid was added to a 50 ​mL vial containing 16 ​mL of water, followed by an injection of 100 ​μL of 1 ​M NaOH. After that, 160 ​μL of HAuCl_4_ (2%) was combined. Then, 4% of CeCl_3_ compared to the molar ratio of gold was combined with the reacting mixture before the dropping of NaBH_4_. The mixture was stirred for 5 ​min. Next, 200 ​μL of 100 ​mM NaBH_4_ was dropped gradually. The mixture stayed for another 24 ​h. Next, HCl (1 ​M) was drooped in the mixture until a turbid was formed. The mixture was centrifugated at 6000 ​rpm and the precipitates were collected and washed with PBS (pH 7.4 buffer). After the precipitate was resuspended in PBS buffer, 0.1 ​M NaOH solution was slowly dropped until the mixture became transparent. The products were monitored by HR-TEM and TEM-EDS. S-DHLA-AuNCs-Ce were synthesized by the same method except S-lipoic acid was used in the place of R-lipoid acid.

### 2.3 Clinical investigation of RA

The data was collected from 73 anti-CCP positive advanced-stage RA patients who were admitted to the First Affiliated Hospital of Jinzhou Medical University, from May 2018 to August 2021 and were selected according to the standards below: The cases meet the European League Against Rheumatism (EULAR) 2010 classification criteria for RA stages; The patients with symptoms of advanced-stage RA were diagnosed by a rheumatologist from the Affiliated Hospital of Jinzhou Medical University; The baseline presence of clinically detected advanced-stage RA was confirmed by the rheumatologist; Symptoms in some patients were relieved by the use of disease-modifying antirheumatic drugs (DMARDs). Corresponding blood analyses were assessed before and after symptom change to compare the relationship of different parameters with RA development. Eligible participants were recruited to a single-center research clinic as part of a prospective observational cohort. The clinical endpoint was the development of RA on clinical examination. All participants provided informed consent for the study before recruitment. The study was approved by the Ethics Committee of the First Affiliated Hospital of Jinzhou Medical University.

### 2.4 Animal preparation and experiments

The animal experiments were conducted in compliance with the guidance for the care and use of laboratory animals and were approved by the institutional biomedical research ethics committee of Jinzhou Medical University. Male SD rats (180–220 ​g, 8–12 weeks) were maintained under specific pathogen-free (SPF) conditions at the Animal Care of Jinzhou Medical University.

Freund's adjuvant and type II collagen were used for emulsion immunization in an arthritic (CIA) model. The rats were immunized with bovine collagen type II emulsion in incomplete Freund's adjuvant and received bovine collagen type II in incomplete Freund's adjuvant 14 days after the first injection. After the second injection of CIA (day 28), the rats were randomly divided into groups (n ​= ​3 rats/group). The severity of CIA was assessed by the thickness of paw swelling. Clinical scores were assessed at the end of the study. After the second injection, the severity of arthritis was scored 3 times a week. In vivo experiments were performed in rats (n ​= ​3 rats/group) 15 days after the second injection.

### 2.5 Histopathological staining

After 15 days of treatment, the joints of rats were amputated and decalcified for one week at 4 ​°C in a solution with 14% ethylenediaminetetraacetic acid (pH 7.2–7.4). For HE staining, sections were immersed in hematoxylin solution for 5 ​min and sluiced in running water for 10 ​s. The sections were differentiated in HCl/95% alcohol (1:50) solution for 10 ​s. The tissues were restained with eosin and then fixed with neutral balsam after dehydration via 75% alcohol, 95% alcohol, and 100% alcohol and transparency with xylene. For Masson staining, sections were dewaxed to water and used Masson compound staining solution for 5 ​min. The tissues were differentiated with acidic ethanol differentiation solution, stained with Masson blue solution, Ponceau magenta staining solution, weak acid working solution, phosphomolybdic acid solution, and aniline blue staining solution for 3 ​min. The tissues were dehydrated via 95% and 100% alcohol, and transparency with xylene, followed by fixing with neutral balsam. For immunofluorescent analysis, sections were blocked with 5% normal goat serum for 1 ​h and incubated overnight at 4 ​°C with primary antibodies. The next day, the tissues were rewashed with PBS and incubated with the secondary antibody at room temperature for 2 ​h. All cell number counts and densities were evaluated by ImageJ software.

### 2.6 Construction and evaluation of CIA rats

CIA rats were modeled and injected twice (days 0 and 14) by the intradermal immunization with incomplete Freund's adjuvant (IFA) containing type II bovine collagen. The arthritis severity was assessed by the clinical scores of paw swelling and scored three times. The clinical score is shown in Table 1.

**Table 1:** Clinical score of paw swelling.

Clinical scores	Symptoms
0	Normal
0.5	Erythema and edema in only one digit
1	Erythema and mild edema of the footpad, or ankle or two to five digits
2	Erythema and moderate edema of two joints (footpad, ankle, two to five digits)
3	Erythema and severe edema of the entire paw
4	Reduced swelling and deformation leading to incapacitated limb

### 2.7 Cell culture and experiments

The cell lines of human fibroblast synovial cells (HFLS), human peripheral blood lymphocyte (RPMI1788) (typical B cells), and embryo osteoblast precursor cells (MC3T3-E1) were maintained in DMEM supplemented with 10% FBS and 1% penicillin/streptomycin. These adherent cell lines were cultured in a humidified atmosphere containing 5% CO_2_ at 37 ​°C and removed from the plastic substrate using a 0.25% trypsin/EDTA solution for passage and before evaluation. For MTT, cells were incubated in 96-well plates in the medium (DMEM supplemented with 10% FBS and 1% penicillin/streptomycin) for 12 ​h. Then the medium was cultured with IL-1β for 1 day, following by various AuNCs (R-DHLA-AuNCs-Ce, R-DHLA-AuNCs, S-DHLA-AuNCs-Ce and S-DHLA-AuNCs) and DHLA (S-DHLA and R-DHLA) and incubated in 5% CO_2_ at 37 ​°C for 1 day. Next day, we added 150 ​μL DMSO for detecting cell viability followed by adding MTT solution to the medium for 6 ​h. For immunohistochemistry, the cells were fixed with a 4% (v/v) buffered formalin solution and immunostained with the anti-TNF-α antibody Alexa Fluor 488. Fluorescence images were obtained using a confocal laser microscope (CLSM, Leica TSCSP5 confocal unit).

### 2.8 Enzyme-linked immunosorbent (ELISA) assays

After 15 days of drug injection to the rats, the cytokine levels in the serum, cartilage, and synovial fluid of the collected samples were analyzed according to the manufacturer's instructions. The absorbance (450 ​nm) was measured using a microplate reader.

### 2.9 RT-qPCR

The samples were collected from the time point for the experiment of RT-qPCR. The relative expression levels of the target genes were normalized to those of the housekeeping gene β-actin and the target genes from the experimental group were compared with the corresponding target genes from the control group using the (1 ​+ ​e)^−ΔΔCT^ method. The following oligonucleotide primers were listed in Supplementary Table 1.

### 2.10 Statistical analysis

Data were expressed as mean ​± ​SD and analyzed by SPSS 23.0. At least three duplicate samples were tested in all experiments. Student's t-test and one-way ANOVA were used to compare the data of two groups and more groups. In addition, the clinical scores were analyzed using the Mann-Whitney *U* test. Wilcoxon's rank-sum test was used to compare the medians of data from the RT-qPCR analysis and histogram analysis. Differences were considered statistically significant with a value of P ​< ​0.05.

## 3 Results

### 3.1 The enhanced B cell abnormalities in advanced-stage RA cases

**Fig. 1:**
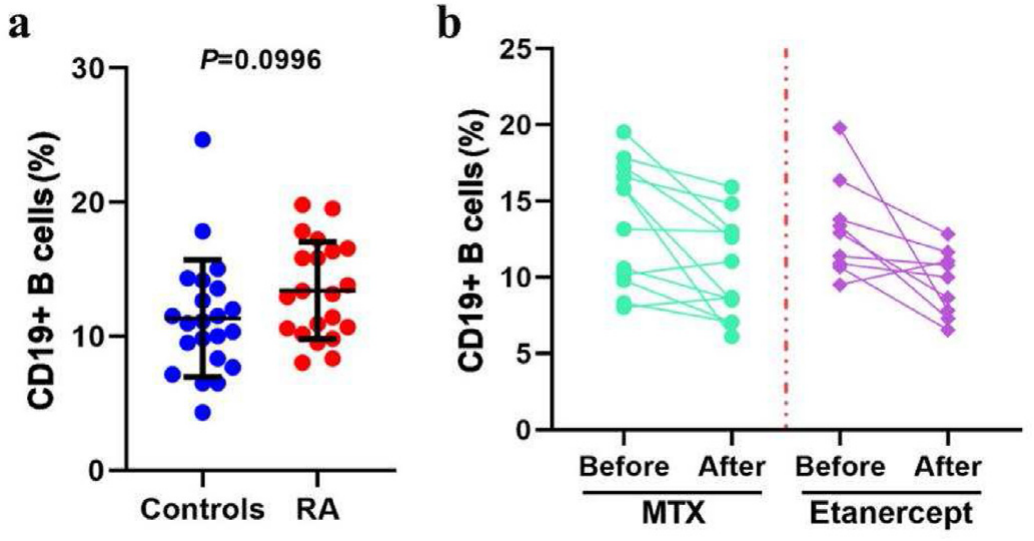
Peripheral blood CD19^+^ B cell levels predict disease progression and drug response in advanced-stage RA cases. (a) Frequency of total CD19^+^ B cells in blood collected from healthy volunteers or patients with advanced-stage RA. (b) Frequency of total CD19^+^ B cells in RA patients before and after treatment with MTX or etanercept for an average period of 12 months.

First, disease-modifying anti-rheumatic drugs (DMARDs) were used to alleviate RA symptoms. We evaluated the efficacy of the DMARDs by different parameters (Supplementary Table 2). Of 73 enrolled patients, 21 patients were defined as advanced-stage RA cases and they were selected for further study. Etanercept and methotrexate (MTX) were therapeutics approved for relieving the symptoms of RA. 9 patients were assigned to the etanercept group and 12 patients were assigned to the MTX group. CD19 is an important biomarker for B cells. Interestingly, we found CD19 was not significantly changed for advanced-stage RA patients compared to the healthy controls (Fig. 1a). However, CD 19 decreased significantly in the patients whose symptoms were relieved by etanercept or MTX treatment (Fig. 1b). This indicates that B Cell balance has a close relationship with the recovery of RA symptoms. In addition, B cells are known to produce rheumatoid factor (RF) mediated production of anti-CCP [1]. They stimulate the secretion of pro-inflammatory cytokines such as TNF-α, which promote inflammation and further worsen RA [20]. The reduced CD19 also indirectly indicated an improvement in inflammation in RA conditions. Binary logistic regression models of the prediction of drug efficacy using, model-1 clinical parameters (red line) and the combined CD 19+ B cells model-2 (blue line) were constructed. The area under the receiver operating characteristic curve (ROC) for the predicted probability of progression from model-1 was 0.64, which represents an improvement over model-2 (0.76) (Supplementary Figure 1). Then, we proposed that CD19^+^ B cells combined with the traditional clinical diagnosis can infer the therapeutic effect of a drug. On the other hand, it can also help in classifying the development of RA after treatment.

### 3.2 Characterization of modified gold nanoclusters (AuNCs)

**Fig. 2:**
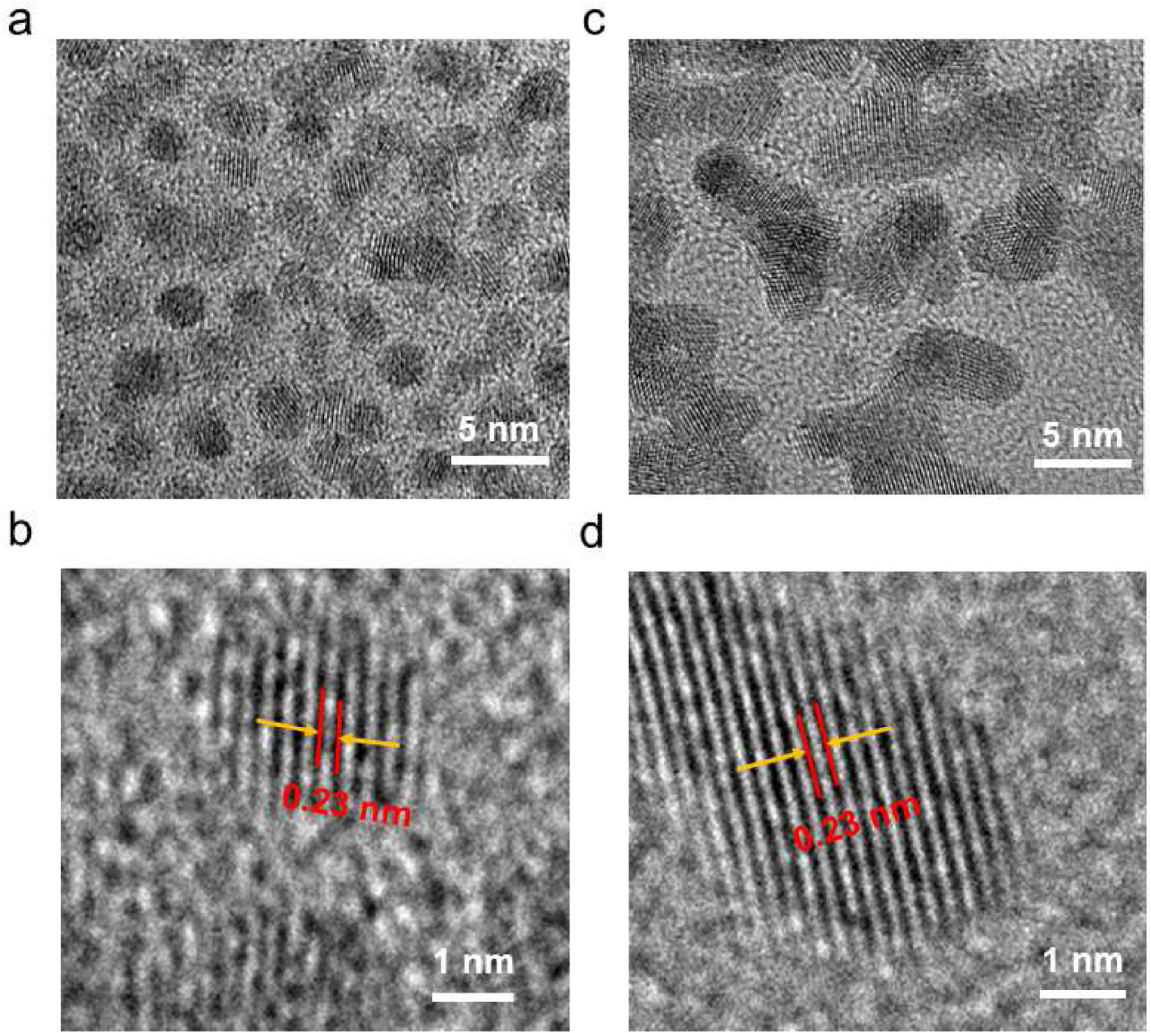
HR-TEM of S-DHLA-AuNCs-Ce (a) and R-DHLA-AuNCs-Ce (c); The figures (a, c) are enlarged to show the lattice fringes of the samples of S-DHLA-AuNCs-Ce (b) and R-DHLA-AuNCs-Ce (d) more prominently.

Firstly, small-sized drugs do not affect the normal function of cells. Secondly, AuNCs have the potential to cross the organ barrier to reach the lesion [21]. Herein, we expect to use the ultra-small AuNCs-based drug to regulate B cells that will indirectly influence acquired immune response. Thereafter, the immunochemotherapy of RA may be achieved (Scheme 1). The morphologies of candidate AuNCs-based drugs including S-DHLA-AuNCs-Ce and R-DHLA-AuNCs-Ce were first carried out by HR-TEM (Fig. 2). For both S-DHLA-AuNCs-Ce (Fig. 2a-b) and R-DHLA-AuNCs-Ce (Fig. 2c-d), an interplanar distance of 0.23 ​nm was observed, which belongs to the planes of gold. This result implied the formation of AuNCs. The content of Ce in the actual samples determines that the S-DHLA-AuNCs-Ce and R-DHLA-AuNCs-Ce products have different sizes. The average size of R-DHLA-AuNCs-Ce is 3.4 ​nm, which is larger than S-DHLA-AuNCs-Ce of 2.4 ​nm. This indicates that more amounts of Ce were attached to R-DHLA-AuNCs. In addition, the dark and light gray areas in the sample can be seen in Fig. 2c and d. The dark gray area is likely due to the stacking of the two NCs. On the other hand, our previous study found that R-DHLA stabilized on the surface of NCs enabled a large number of carboxyl groups present [19]. When the Ce content increased, two or more NCs could be cross-linked, which facilitated the formation of assembled samples. However, when the Ce content is less than 4%, the sample is still in a dispersed and water-soluble state, and further injection application can be performed. Moreover, the elements in and around the AuNCs were detected by energy-dispersive X-ray spectroscopy (EDS) (Supplementary Figure 2). Both peaks of Au and Ce were observed in R-DHLA-AuNCs-Ce samples, but no obvious peak was observed in S-DHLA-AuNCs-Ce samples. Thus, compared to S-DHLA stabilized AuNCs, Ce is more successfully modified on R-DHLA-AuNCs. To further analyze the composition of R-DHLA-AuNCs-Ce, we performed an EDS-Mapping analysis (Supplementary Figure 3). The EDS-Mapping of particles smaller than 5 ​nm could not be observed, so we investigated the elemental distribution of multiple R-DHLA-AuNCs-Ce at smaller magnifications. The distribution range of Ce element and Au element is comparable, which further indicates the effective modification of Ce on R-DHLA-AuNCs. We also use ICP-MS to confirm that larger than 2% of Ce were always present in R-DHLA-AuNCs-Ce by repeating the synthesis methods. Since the synergistic effect of AuNCs and Ce were expected, we only conducted experiments with R-DHLA-AuNCs-Ce for RA treatment.

### 3.3 R-DHLA-AuNCs-Ce suppress B cell-mediated acquired immunity in vitro

**Fig. 3:**
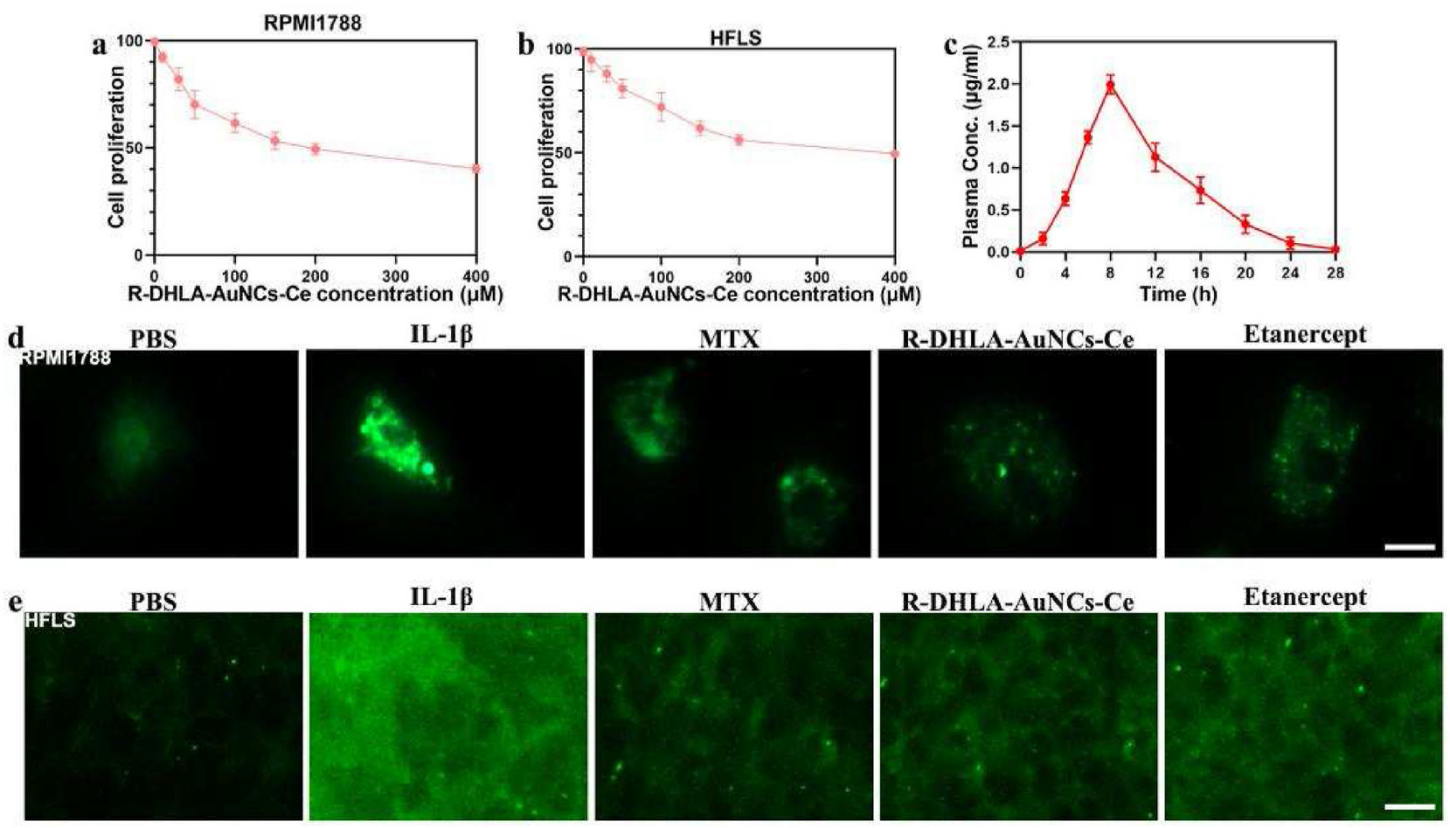
Biological functions and anti-inflammatory effects of R-DHLA-AuNCs-Ce. (a) Schematic illustration of the use of R-DHLA-AuNCs-Ce for the treatment of RA. Inhibition curves of R-DHLA-AuNCs-Ce-treated RPMI1788 (b) and human fibroblast synoviocytes HFLS (c). (d) In vivo plasma concentration profile of R-DHLA-AuNCs-Ce. Immunofluorescence staining of TNF-α in R-DHLA-AuNCs-Ce-treated RPMI1788 (e) and HFLS (f). The scale bar is 25 ​μm.

According to our previous studies, R-DHLA-modified AuNCs and their derived materials have better anti-oxidative stress ability than S-DHLA-modified AuNCs [19]. This is likely to make R-DHLA-modified AuNCs more effective in many ‘Reactive oxygen species' ROS-related diseases. However, for advanced-stage of RA, the greatest need for suppression is chronic inflammation. Therefore, we first initially investigated the anti-inflammatory responses of the two different AuNCs-based drugs in vitro. IL-1β was used as a marker and stimulator to induce human peripheral blood lymphocytes (RPMI1788) and human fibroblast synovial cells (HFLS), which provided excellent indicators for the development of joint diseases and inflammation [2]. We found that R-DHLA-AuNCs-Ce (100 ​μM) could more significantly reduce the number of RPMI1788 and HFLS based on the MTT analysis than S-DHLA-AuNCs-Ce (100 ​μM) (Supplementary Figure 4), indicating R-DHLA-AuNCs-Ce was potentially more efficient to treat RA. In our previous work [19], both Ce^3+^ and Ce^4+^ were found to exist in R-DHLA-AuNCs-Ce. The charge transfer from Ce^3+^ to Ce^4+^ can reduce ROS levels, which subsequently influence cytokine levels. We found Ce modified AuNCs could more efficiently regulate the cytokines such as TNF-α than AuNCs. These cytokines were supposed to boost the immune response of B cells but were suppressed. Therefore, Ce modified AuNCs more efficiently inhibit the immune response of B cells than AuNCs, making the immune efficacy much better. The inhibition efficiency of RPMI1788 and HFLS increased as a function of R-DHLA-AuNCs-Ce, reaching a half value at 100 ​μM. (Fig. 3a and b). Then, 100 ​μM of R-DHLA-AuNCs-Ce that showed excellent inhibition effects were used for further treatment.

To investigate if R-DHLA-AuNCs-Ce is superior to traditional RA drugs for symptom relief, we compared it with clinical drugs including MTX and etanercept. In the PBS control group, limited cell proliferation was observed (Supplementary Figure 5a). On the other hand, proliferation was significantly increased when RPMI1788 was treated with IL-1β. All drugs, including MTX, etanercept, and R-DHLA-AuNCs-Ce, showed inhibitory effects on IL-1β-induced lymphocyte proliferation (Supplementary Figure 5a). However, the R-DHLA-AuNCs-Ce group and the etanercept group showed better inhibition efficiency. Proliferating fibroblast synovial cells exacerbated the progression of advanced RA. Herein, we observed an IL-1β inhibitory effect for human fibroblast synovial cells (HFLS) (Fig. 3c, Supplementary Figure 5b).

To investigate the residual drug after administration of R-DHLA-AuNCs-Ce and the drug circulation, the plasma content of R-DHLA-AuNCs-Ce was investigated in CIA model rats (Fig. 3d). The elimination half-life of R-DHLA-AuNCs-Ce was shortened to 28 ​h, compared with MTX (over 48 ​h) and etanercept (over 70 ​h), indicating that R-DHLA-AuNCs-Ce can be washed more effectively after medication. As shown in Fig. 3e,f, and Supplementary Figure 6, MTX, etanercept, and R-DHLA-AuNCs-Ce all reduced TNF-α levels in RPMI1788 and HFLS. However, only R-DHLA-AuNCs-Ce down-regulated the IL-6 level more significantly (Supplementary Figure 7). These results suggest that R-DHLA-AuNCs-Ce had a better overall anti-inflammatory effect in vitro. The osteogenic differentiation of MC3T3-E1 was evaluated by immunofluorescence staining, Alizarin Red staining (ARS), and RT-PCR. R-DHLA-AuNCs-Ce more efficiently improved the morphology of IL-1β-induced cells (Supplementary Fig. 8a-8d). The formation of the mineralized nodules was tested by ARS on day 21, by which calcium nodules can be stained red (Supplementary Figure 7e). The gene expressions of bone-related mRNA (Runx2, BMP-2, OCN, and OPN) (Supplementary Figure 9) were also detected. All osteogenic markers (Runx2, BMP-2, OCN, and OPN) were upregulated in MC3T3-E1 cells treated with the drugs. However, R-DHLA-AuNCs-Ce and etanercept promoted osteogenic differentiation more efficiently.

R-DHLA-AuNCs-Ce circulates in the body for a short time, but the subsequent therapeutic effect is continued, which reveals that R-DHLA-AuNCs-Ce possibly produced the therapeutic effects by the immune memory. Therefore, we further investigated whether R-DHLA-AuNCs-Ce could influence the activity of B cells. RPMI1788 (B lymphocyte cell line) was incubated with IL-1β in a medium with (R-supernatant) or without (supernatant) R-DHLA-AuNCs-Ce for 24 ​h. Then, the cells were washed thoroughly with PBS and incubated for an additional 24 ​h. After that, the supernatant was collected by centrifugation and then co-cultured with other IL-1β-treated RPMI1788 ​cells (IL-1β/R-supernatant and IL-1β/supernatant). After 24 ​h of incubation, the expression of TNF-α was assessed by confocal microscopy. TNF-α levels were significantly downregulated by IL-1β/supernatant (Supplementary Figure 10). This indicated that R-DHLA-AuNCs-Ce significantly suppressed B cell-mediated adaptive immunity and achieved enhanced anti-inflammatory effects in vitro.

### 3.4 Symptom recovery in CIA rats

**Fig. 4:**
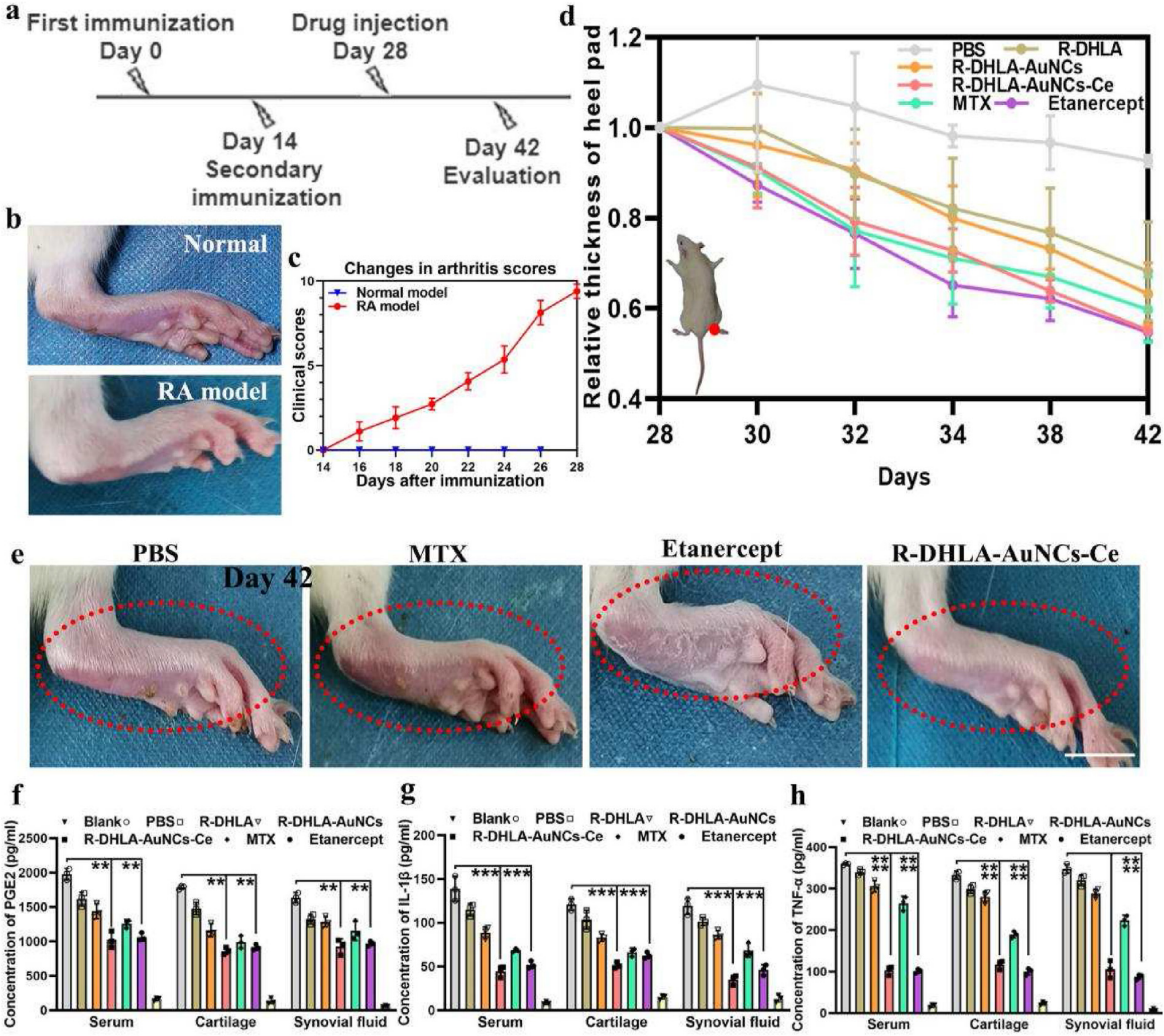
Symptom recovery in a rat model of CIA. (a) Timeline of the in vivo bioefficacy assay. (b) Comparison of healthy rat paws with established advanced-stage RA model paws. (c) Clinical score of the RA model as a function of immunization time. (d) Relative thickness plot of heel pads treated in different ways over time. (e) Representative photographs of rat heel pads treated with different drugs. Quantitative analysis of PGE2 (f), IL-1β (g), and TNF-α (h) levels in different treatment groups. The scale bar is 1 ​cm ∗∗p ​< ​0.01; ∗∗∗p ​< ​0.001; ∗∗∗∗p ​< ​0.0001.

To systematically investigate the drug efficacy of R-DHLA-AuNCs-Ce, we used a secondary immunization approach to maximally induce RA in rats to achieve advanced-stage symptoms (Fig. 4a). A rat model of advanced CIA was successfully established by conventional diagnosis and evaluation of CD19 biomarkers (Fig. 4b). In addition, two blinded clinical assessments were performed to quantitatively assess rats as blank controls. Rats with advanced-stage RA scored much higher than unimmunized controls (Fig. 4c). We then investigated the changes in rat heel pad thickness over time (Fig. 4d; Supplementary Figure 11). The symptoms were significantly relieved 10 days after R-DHLA-AuNCs-Ce injection. The recovery is much faster than most drugs for RA symptom relief [17,[22], [23], [24], [25], [26], [27]]. The characteristic contrast photos of heel pads treated with different agents further revealed the superior therapeutic effect of R-DHLA-AuNCs-Ce in advanced RA rats (Fig. 3e). Compared with the PBS, MTX, and etanercept groups, after R-DHLA-AuNCs-Ce treatment, the joint morphology was closer to the healthy state, showing a smoother surface and a more complete structure.

Typical cytokines in advanced-stage RA rats, including IL-1β, PGE2, and TNF-α, were quantified by ELISA. On day 42, specimens were collected from serum, cartilage, and synovial fluid from control rats and RA rats treated with R-DHLA-AuNCs-Ce, etanercept, MTX, and PBS, respectively (Fig. 4f-h). R-DHLA-AuNCs-Ce reduced pro-inflammatory cytokines more significantly than MTX. On the other hand, there was no significant difference between the etanercept and R-DHLA-AuNCs-Ce groups. This suggests that both R-DHLA-AuNCs-Ce and etanercept induced stronger immunity, resulting in better recovery from RA-induced symptoms than MTX.

### 3.5 Tissue regeneration in CIA rats

**Fig. 5:**
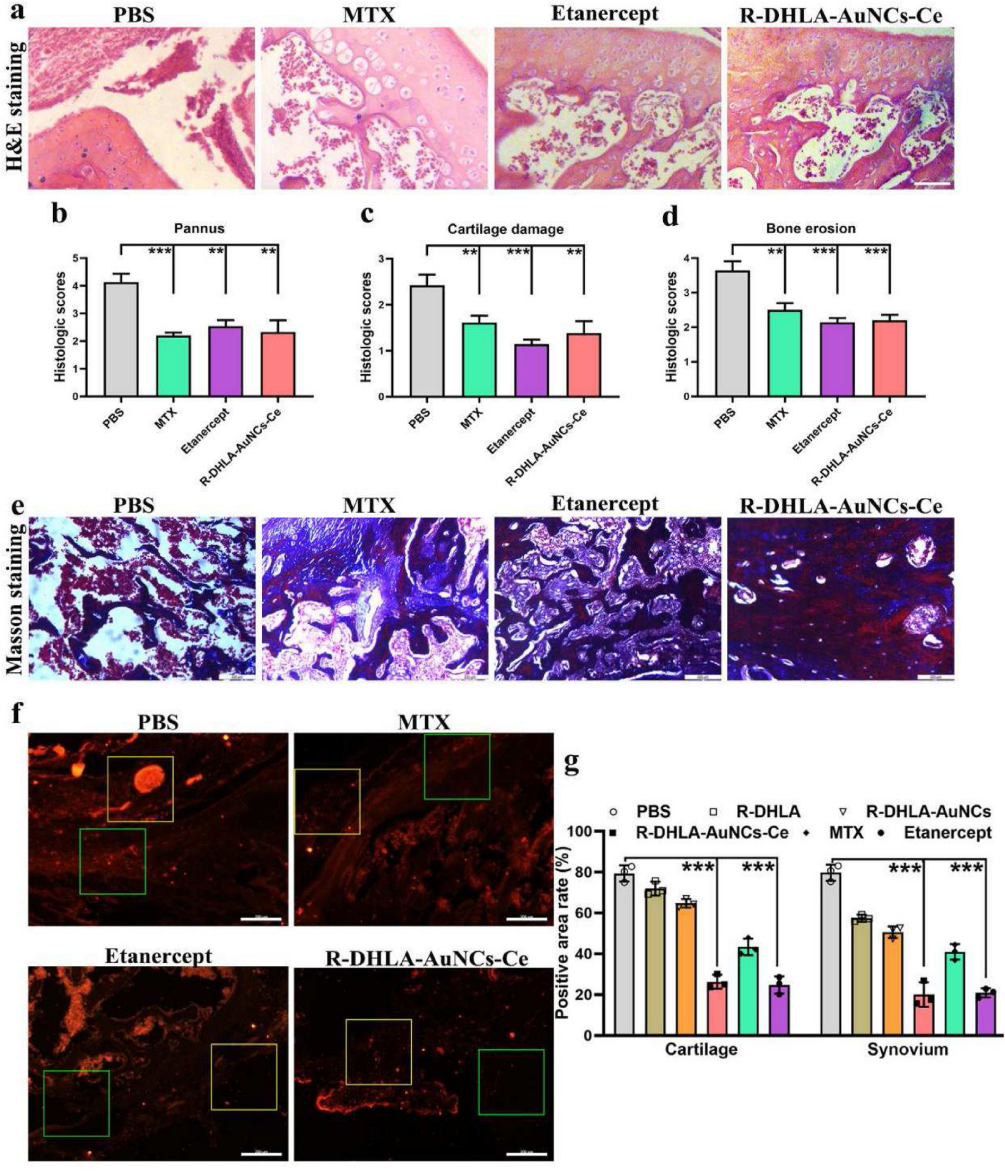
Histological sections and immunohistochemical analysis of joint tissue. Bone erosion protocol in CIA rats (a). Hematoxylin and eosin staining (b) and the number of pannus (c), cartilage (d), and bone (e) of joint tissue in different groups. Masson staining of joint tissue in different groups (f). Images (g) and quantitative results (h) of TNF-α immunofluorescence in cartilage and synovial sections. Green boxes represent synovial tissue and yellow boxes represent cartilage. The scale bar is 200 ​μm. Statistical analysis was determined using a two-tailed *t*-test, ∗∗∗p ​< ​0.001; ∗∗∗∗p ​< ​0.0001.

To further investigate the therapeutic effect of the drug, H&E, Masson, and immunofluorescence staining were investigated. The H&E staining results of the R-DHLA-AuNCs-Ce, etanercept and MTX groups showed that the cartilage area was smooth and complete, while the PBS group showed a rough and irregular pattern, indicating that the cartilage tissue damage in RA rats was not alleviated until the administration of effective drugs (Fig. 5a-d). On the other hand, only R-DHLA-AuNCs-Ce significantly reduced bone erosion (Fig. 5e). MTX and etanercept were less effective at repairing bone. The proinflammatory cytokine TNF-α, which plays a key role in the pathophysiology of RA, was investigated by immunofluorescence staining. Compared with PBS and MTX groups, TNF-α levels in cartilage and synovial tissue were more significantly decreased in the R-DHLA-AuNCs-Ce group (Fig. 5f-g). By comprehensive comparison, R-DHLA-AuNCs-Ce was the most effective drug in restoring the healthy state of joint tissues. Compared with RA drugs investigated in the references (Table 2), the currently developed cluster drugs have great potential to more rapidly cure (10 days) advanced-stage RA.

**Table 2:** Comparison of the recovery of CIA animal models by using different therapeutics.

Therapeutics	Arthritis recovery	Targets	Advantages/*Disadvantages*	Ref.
GANT61	improve significantly after 2 weeks	fibroblast	promotes fibroblast apoptosis; ***soluble in ethanol only***	[26]
Glucocorticoids	improve significantly after 30 days	Bone	reduces inflammation; ***water-sodium retention, susceptible to infection.***	[27]
MTX	improve significantly after 13 days	CD4^+^ T cells	reduces inflammation; ***long-term toxicity, myelosuppression***	[25]
FA-AgNPs	improve significantly after 20 days	macrophages	promotes macrophage polarization; ***toxic concern***	[17]
MNP	improve significantly after 2 weeks	vessels	inhibit pannus formation; ***complex process ​of ​synthetic ​route***	[24]
Etanercept	improve significantly after 2 weeks	dendritic cell	Symptom relief; ***long-term toxicity***	[24]
R-DHLA-AuNCs-Ce	improve significantly after 10 days	B cells, fibroblast, bone	Remarkable treatment effects, low toxicity, cost-effective, efficiently clearance	current work

Note: FA-AgNPs, folic acid-modified silver nanoparticles; MNP, macrophage-derived microvesicle-coated nanoparticle.

### 3.6 Biocompatibility of R-DHLA-AuNCs-Ce in vivo and in vitro

**Fig. 6:**
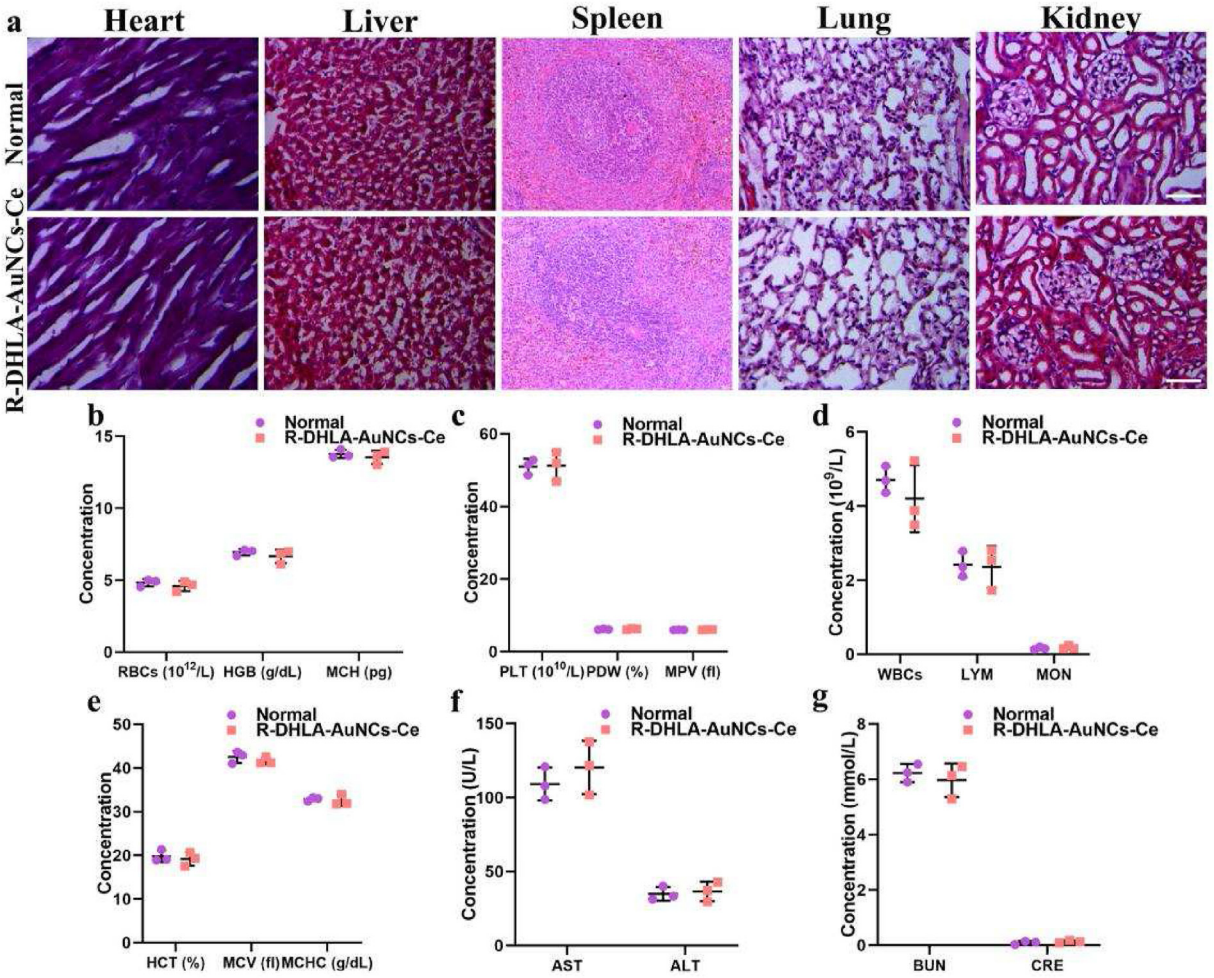
In vivo biocompatibility of R-DHLA-AuNCs-Ce. (a) In vivo toxicity of R-DHLA-AuNCs-Ce to major organs (heart, liver, spleen, lung, and kidney) was assessed 30 days after intravenous administration. (b–e) Blood parameters of healthy rats (control group) and the rats after intravenous injection of R-DHLA-AuNCs-Ce for 30 days. Serum AST, ALT (f), BUN, and CRE (g) levels in healthy rats (control group) and the rats after intravenous injection of R-DHLA-AuNCs-Ce for 30 days.

We assessed drug safety by evaluating the effects of R-DHLA-AuNCs-Ce (300 ​μM, 3-fold higher concentration than CIA rats) on histopathology and blood chemistry of major organs in healthy rats. As shown in Fig. 6a, no necrosis, congestion, or hemorrhage was observed in the heart, liver, spleen, lung, and kidney 30 days after daily intravenous injection of R-DHLA-AuNCs-Ce. Platelet analysis (Fig. 6b-e) showed no significant difference in hematology in the R-DHLA-AuNCs-Ce-treated group compared with the control group. Serum biochemical analysis (Fig. 6f-g) showed that liver function indicators (aspartate aminotransferase (AST) and alanine aminotransferase (ALT)) and renal function indicators (BUN and CRE) in R-DHLA-AuNCs-Ce were normal. The serum concentration of the treated group was similar to that of the control group and showed excellent biocompatibility in the liver and kidney. All results confirmed that R-DHLA-AuNCs-Ce exhibited negligible in vivo toxicity. In addition, no significant changes were observed in the fluorescence emission intensity of R-DHLA-AuNCs-Ce following 3 days of storage in PBS or FBS at 4 ​°C, indicating that these particles were highly stable (Supplementary Figure 12). Overall, R-DHLA-AuNCs-Ce may have clinical advantages over the most popular RA drugs, such as MTX and etanercept, when biosafety, treatment duration, and treatment efficiency are fully considered.

## 4 Discussion

Strategies to modulate the inflammatory microenvironment to improve RA joint symptoms have been applied in recent years, such as chemotherapy and the administration of biologics. However, these therapies require long-term medication. Accurately distinguishing RA progression is also very difficult. In advanced-stage RA cases, we observed several specific abnormalities in clinical features (Supplementary Table 2), including marked upregulation of anti-CCP, RF, C-reactive protein (CRP), and erythrocyte sedimentation rate (ESR), with exacerbation Joint symptoms. We also observed an increase in B cells in the blood of advanced-stage RA cases. This abnormal distribution of immune/inflammatory cell populations suggests striking differences in systemic immune responses between RA patients with early-stage, advanced-stage, and other diseases (Fig. 1). We can combine complex changes in these parameters as RA biomarkers with traditional diagnosis to improve the classification accuracy of disease stages. In this study, we observed the regulation of pro-inflammatory cytokines expression and lower acquired immunity response in IL-1β-induced B cells treated with R-DHLA-AuNCs-Ce. Moreover, the treatment of R-DHLA-AuNCs-Ce in IL-1β-induced HFLS and MC3T3-E1 cells might also indicate protective therapeutics of RA to present anti-inflammatory and osteogenic differentiation, contributing to RA recovery (Fig. 3 and Supplementary Figure 4-10). We further demonstrated that significantly better curative effect of R-DHLA-AuNCs-Ce in CIA rats (Figs. 4 and 5), compared to MTX and Etanercept. Although the current work is mainly focused on RA immunochemotherapy, these Ce modified AuNCs may be extended to other autoimmune diseases.

## 5 Conclusion

We successfully constructed a noninvasive assessment of advanced-stage RA using biomarkers related to B cells in the blood. Furthermore, R-DHLA-AuNCs-Ce were used to suppress the immune response elicited by B cells and stimulate B cells to produce immune memory. After the end of the treatment, the immune efficacy continued to show anti-inflammatory effects and restore advanced-stage RA symptoms. The current strategy not only brings new hope to patients with advanced-stage RA but also provides a new direction for the safe treatment of other autoimmune diseases.

## Data availability statement

Most of the datasets supporting the conclusions of this article are included within this article. The datasets used or analyzed during the current study are available on reasonable request.

## Credit author statement

K. Gao, D. Li and X. Mei designed the idea of this work. S. Lin supervised the project. W. Gao provided guidance for the diagnosis and data analysis of late RA patients. D. Li and S. Lin conducted the material preparation and cell experiment and analyzed the data. J. Sun performed in vivo experiment and analyzed the data. K. Gao, D. Li and X. Mei wrote the manuscript.

## Declaration of competing interest

The authors declare that they have no known competing financial interests or personal relationships that could have appeared to influence the work reported in this paper.
