# Supplementary material for: Developing cerium modified gold nanoclusters for the treatment of advanced-stage rheumatoid arthritis
Source: Mater Today Bio. 2022 Jun 23;15:100331. doi: 10.1016/j.mtbio.2022.100331 (PMC9251783; doi:10.1016/j.mtbio.2022.100331)
Supplement: Multimedia component 1 [file mmc1.docx]

Supplementary material

**Developing cerium modified gold nanoclusters for the treatment of advanced-stage rheumatoid arthritis**

Sen Lin^a,b^, Wei Gao^c^, Jiachen Sun^a^, Kai Gao^d,*^, Dan Li^e,*^, Xifan Mei^a,b,*^

^a^Department of Orthopedics, Third Affiliated Hospital of Jinzhou Medical University, Jinzhou, P. R. China..

^b^Key Laboratory of medical tissue engineering, Jinzhou Medical University, Jinzhou, P. R. China.

^c^Department of Rheumatology and Immunology, First Affiliated Hospital of Jinzhou Medical University, Jinzhou, P. R. China.

^d^Department of Orthopedics, Jining No. 1 People's Hospital, Jining, P. R. China.

^e^College of Pharmacy, Jinzhou Medical University, Jinzhou, P. R. China.

**^*^Corresponding Author**

Kai Gao, M.D., gaohaikai88@126.com

Dan Li, Ph.D., danli@jzmu.edu.cn

Xifan Mei, M.D., meixifan@jzmu.edu.cn

**This PDF file includes:** Supplementary Figure 1 To Figure 12, Supplementary Table 1 To Table 2.


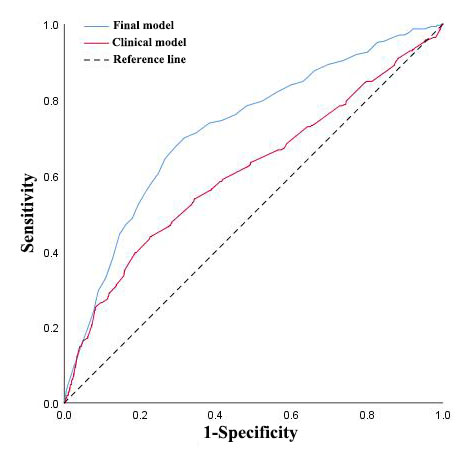


**Supplementary Figure 1.** Receiver operating characteristic curve (ROC) graphical representation of the three logistic regression models. Binary logistic regression models of the prediction of drug efficacy using, model-1 clinical parameters (red line) and the combined CD 19+ B cells model-2 (blue line). CD19+ B cells combined with the traditional clinical diagnosis can infer the therapeutic effect of a drug.


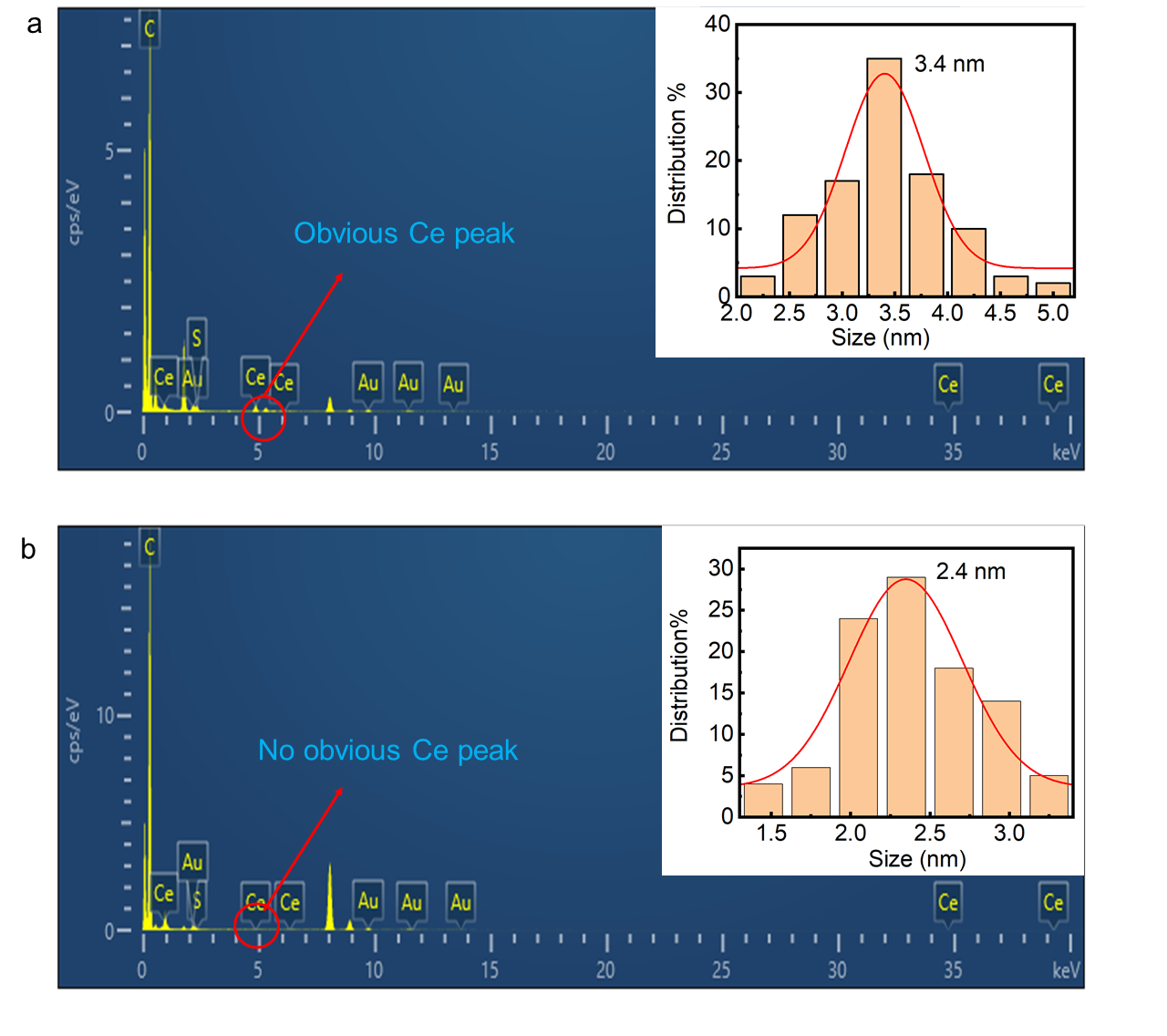


**Supplementary Figure 2.** TEM-EDS of R-DHLA-AuNCs-Ce (a) of S-DHLA-AuNCs-Ce (b); The inset shows the corresponding size distribution of the samples.


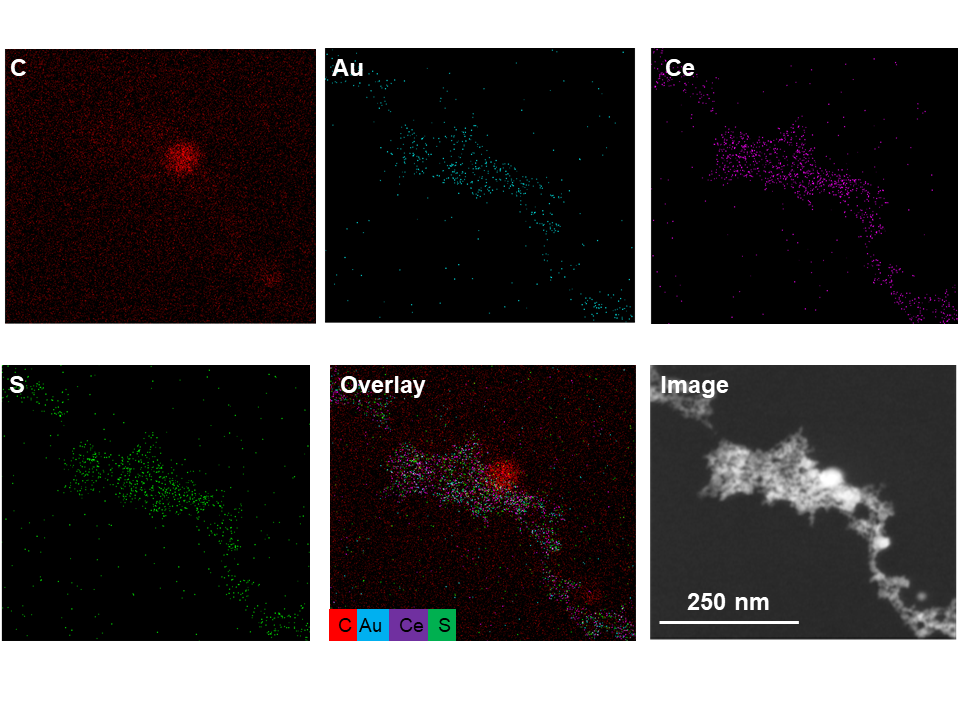


**Supplementary Figure 3.** EDS-Mapping of R-DHLA-AuNCs-Ce


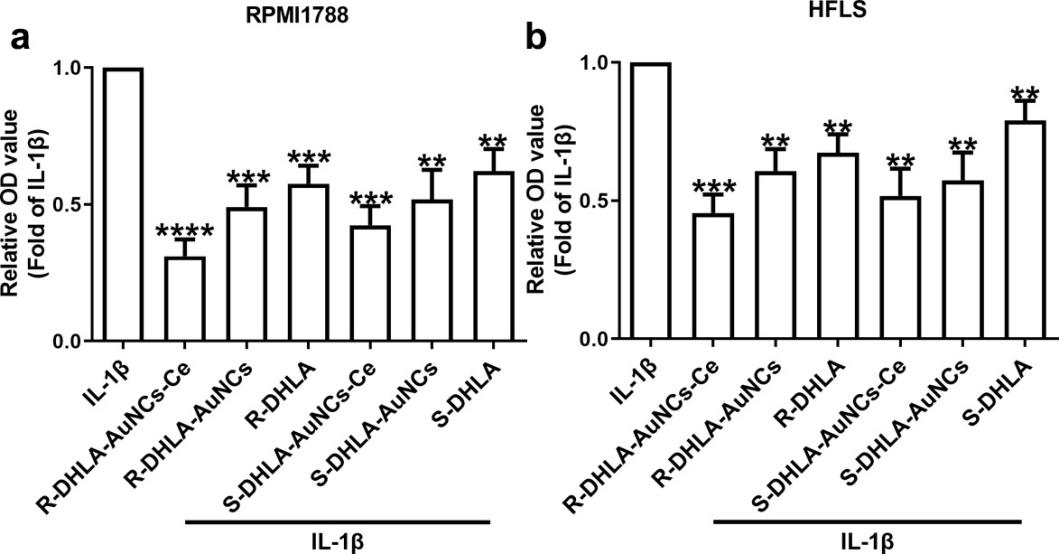


**Supplementary Figure 4.** Cell proliferation of IL-1β-induced human peripheral blood lymphocyte (RPMI1788) and human fibroblast synovial cells (HFLS) treated with various agents including R-DHLA-AuNCs-Ce and S-DHLA-AuNCs-Ce.


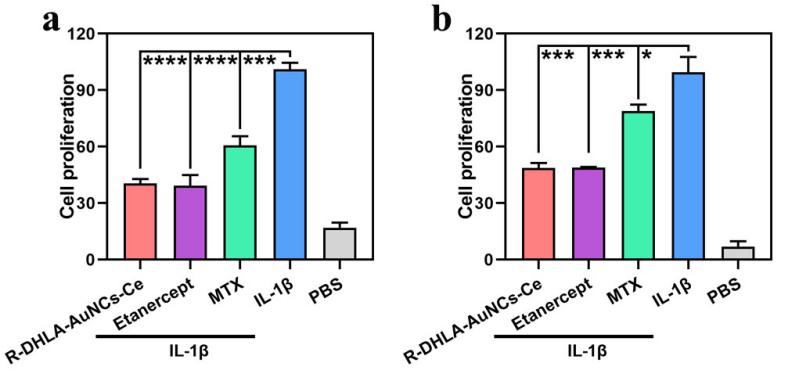


**Supplementary Figure 5.** The inhibition of R-DHLA-AuNCs-Ce in IL-1β-induced human peripheral blood lymphocyte (RPMI1788) and human fibroblast synovial cells (HFLS) by MTT.


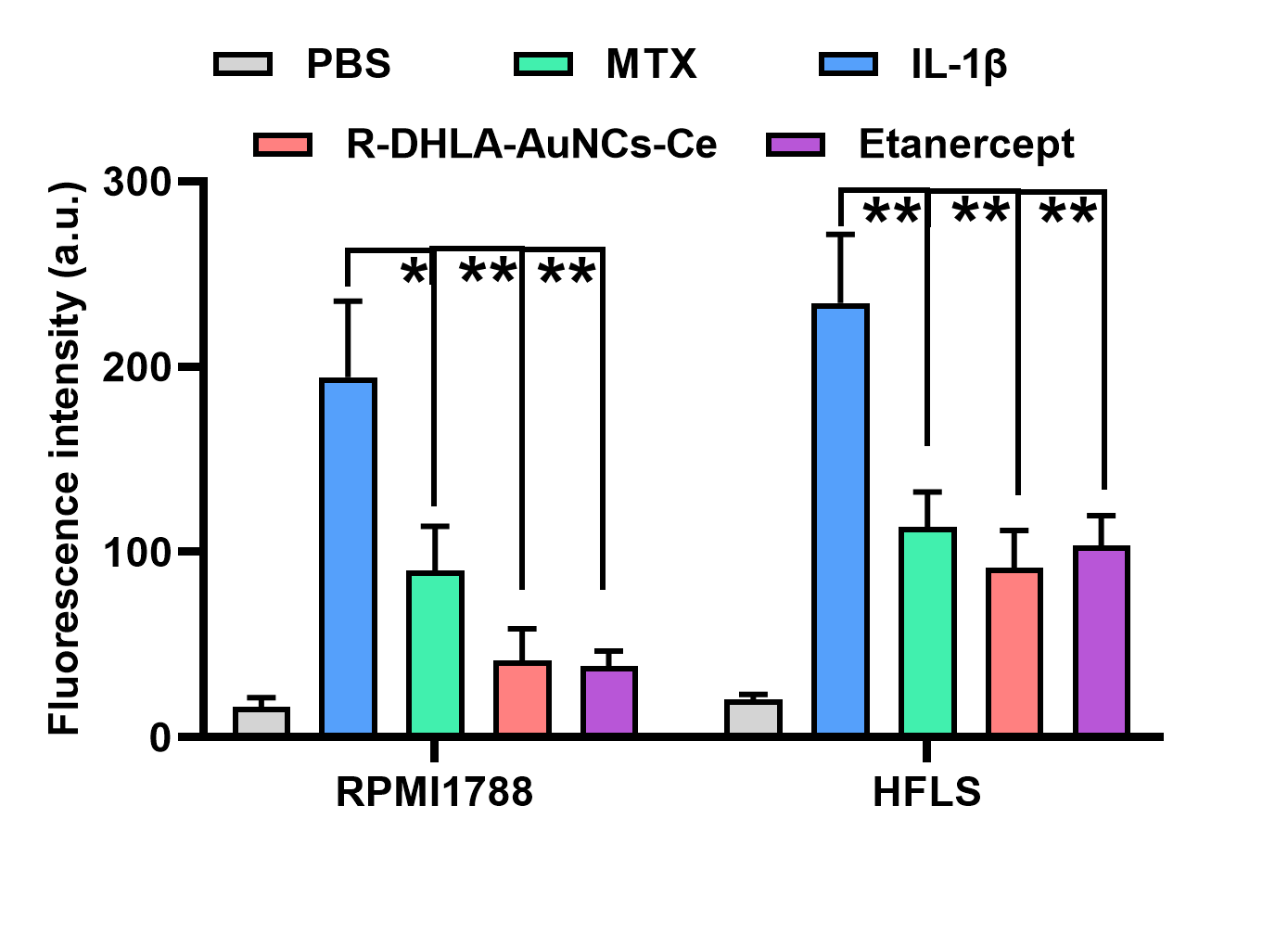


**Supplementary Figure 6.** Fluorescence intensity of TNF-α in human peripheral blood lymphocyte (RPMI1788) and human fibroblast synovial cells (HFLS) after the treatment with different agents.


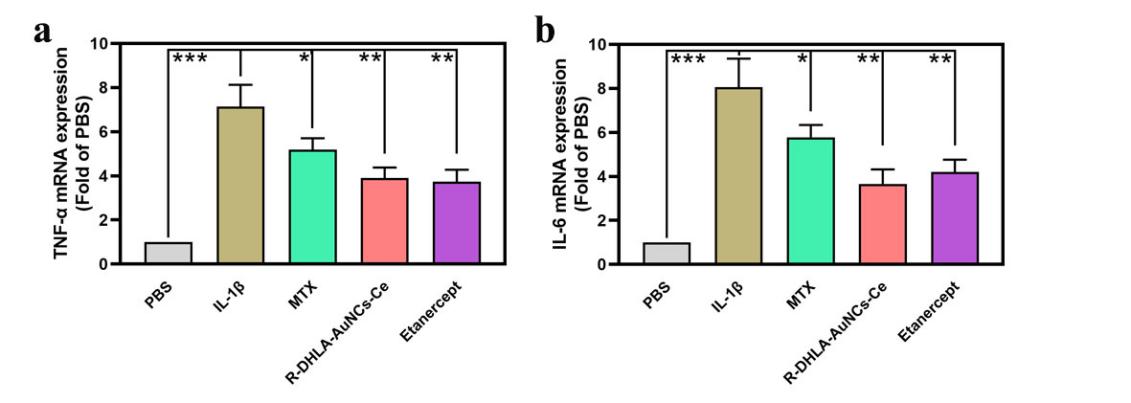


**Supplementary Figure 7.** Downregulation of proinflammatory cytokines in IL-1β-induced HFLS by the treatment of different agents. The mRNA levels of TNF-α (a) and IL-6 (b) were studied by RT-qPCR.


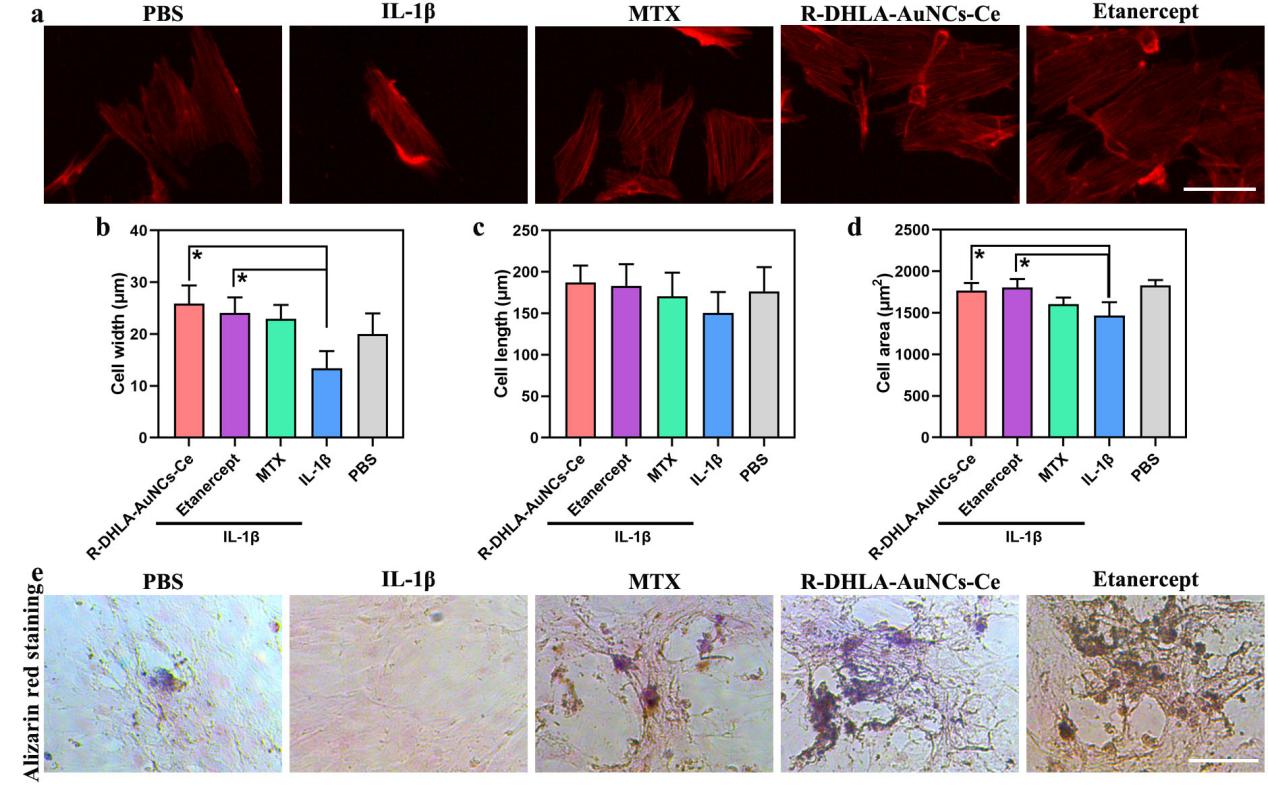


**Supplementary Figure 8**. Osteogenic effects of various treatments on IL-1β-induced MC3T3-E1. (a) The immunofluorescence staining of MC3T3-E1 in different treatment groups. The analysis of cell width (b), cell length (c), and cell area (d) of MC3T3-E1 in different treatment groups. The Alizarin red staining (e) of MC3T3-E1 in different treatment groups.


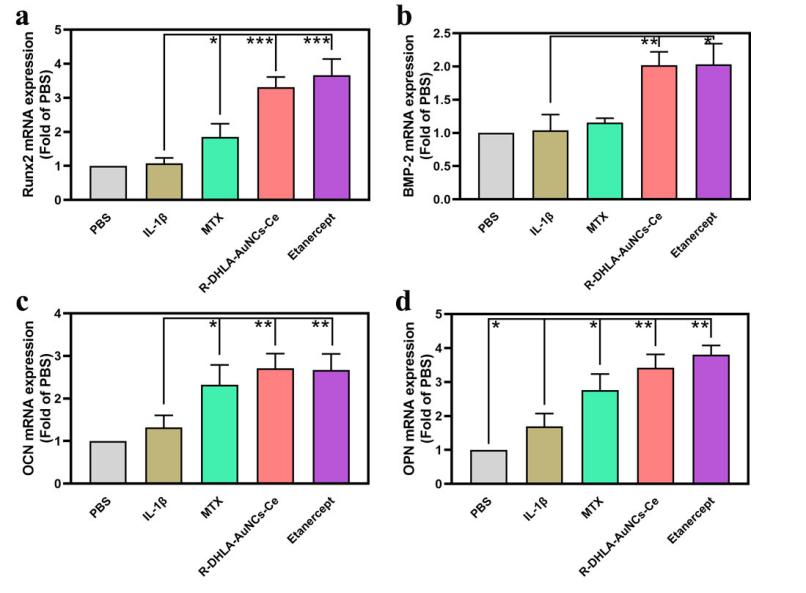


**Supplementary Figure 9.** Osteogenic formation in IL-1β-induced MC3T3-E1 by the treatment of different agents. The mRNA levels of Runx2 (a), BMP-2 (b), OCN (c), and OPN (d) were studied by RT-qPCR


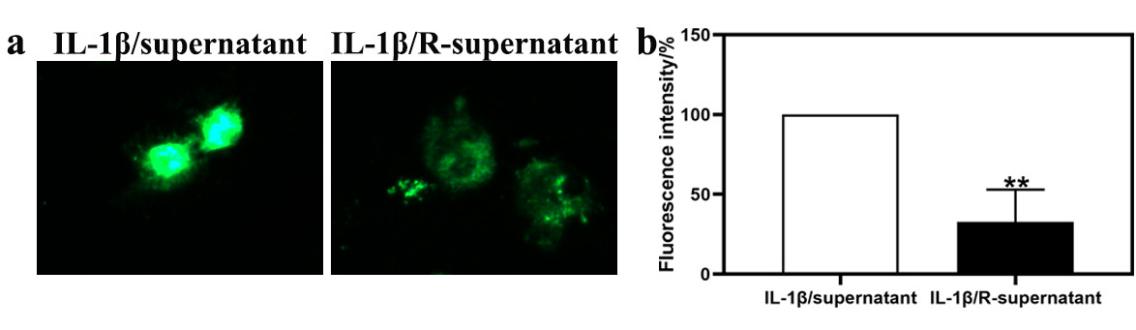


**Supplementary Figure 10.** (a) IL-1β-induced adaptive immune response in RPMI1788. Immunofluorescence staining of TNF-α in RPMI1788 treated with R-DHLA-AuNCs-Ce supernatant. (b) Corresponding semi-quantitative analysis of TNF-α.


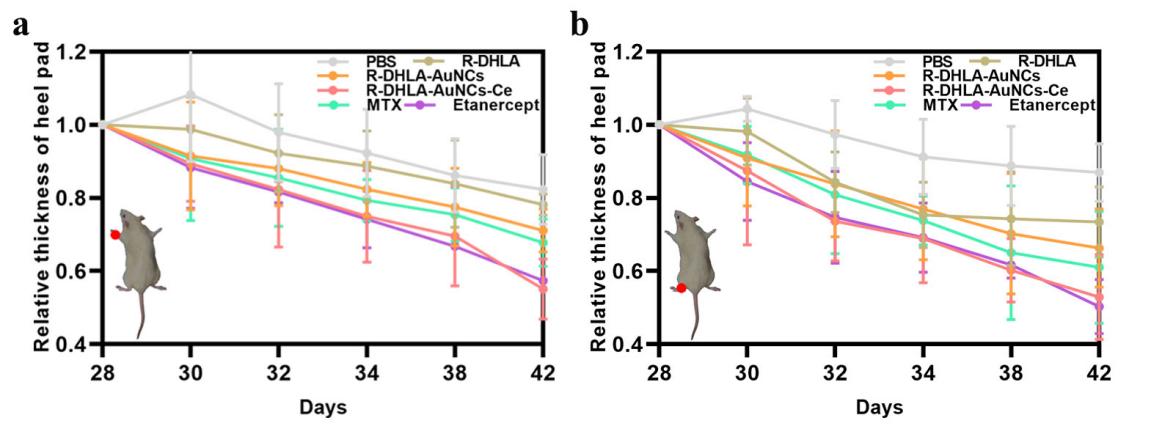


**Supplementary Figure 11.** Heel pad recovery in CIA rats. The plot of the relative thickness of left forelimb (a),

left hindlimb (b) treated with different modalities over time (a, b).

R-DHLA-AuNCs-Ce circulates in the body for less than 28 hours, accompanied by the activation of the immune memory of B Cells, which further fight rheumatoid arthritis. Therefore, the drug needs to be stable for 28 hours in physiological environments. Fluorescence is the most important property of AuNCs. When AuNCs are destroyed, the fluorescence is also likely to be quenched. Therefore, we investigated the fluorescence changes of R-DHLA-AuNCs-Ce in the physiological environment for 48 hours. Herein, we used fetal bovine serum (FBS) at pH 7.4 to mimic the physiological environment, the fluorescence intensity of R-DHLA-AuNCs-Ce initially increased with time, reached a plateau within 10 h, and then the intensity changed insignificantly. R-DHLA-AuNCs-Ce interacted with proteins after dispersed in FBS. The adsorption of proteins on the surface of R-DHLA-AuNCs-Ce enables the surface to become less polar than that dispersed in solution[1]. Then, this changes the surface states which play an essential role in the fluorescence of NCs. However, when the adsorption was stabilized, the fluorescence intensity did not continue to increase. The results showed the excellent stability of R-DHLA-AuNCs-Ce under the physiological environment.


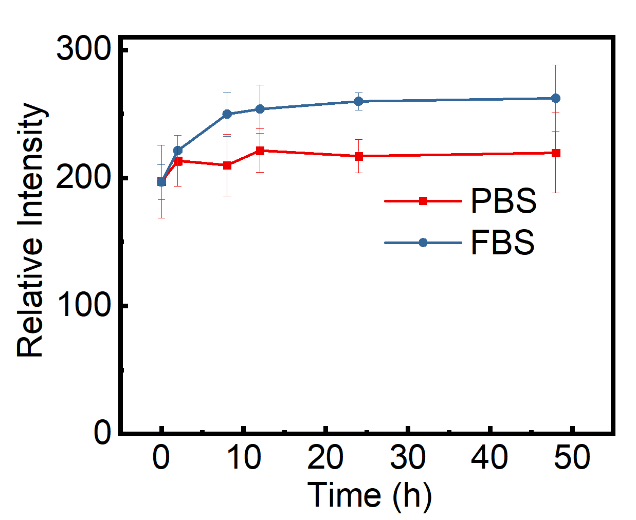


**Supplementary Figure 12.** Relative fluorescence emission intensity at 605 nm of R-DHLA-AuNCs-Ce in PBS and FBS with a 365 nm.

**Supplementary Table 1 Nucleotide sequences of primers used in real-time PCR**

| Gene | Nucleotide sequences 5’-3’ |
| --- | --- |
| TNF-α (Forward) | TAGCCCACGTCGTAGCAAAC |
| TNF-α (Reverse) | TGTCTTTGAGATCCATGCCGT |
| IL-6 (Forward) | ACTCACCTCTTCAGAACGAATTG |
| IL-6 (Reverse) | CCATCTTTGGAAGGTTCAGGTTG |
| Runx2 (Forward) | CCGCCTCAGTGATTTAGGGC |
| Runx2 (Reverse) | GGGTCTGTAATCTGACTCTGTCC |
| BMP-2 (Forward) | GCACTGGGTTGCATTGGTTT |
| BMP-2 (Reverse) | GGGAAGCAGCAACACTAGAAGA |
| OCN (Forward) | GCAAAGGTGCAGCCTTTGTG |
| OCN (Reverse) | GGCTCCCAGCCATTGATACAG |
| OPN (Forward) | TCACCTGTGCCATACCAGTTAA |
| OPN (Reverse) | TGAGATGGGTCAGGGTTTAGC |
| β-actin (Forward) | CGTAAAGACCTCTATGCCAACA |
| β-actin (Reverse) | AGCCACCAATCCACACAGAG |

**Supplementary Table 2 Clinical characterization of advanced-stage RA patients.**

|  | **Controls** | **Etanercept (n = 9)** | | **MTX (n = 12)** | |
| --- | --- | --- | --- | --- | --- |
|  | **(n = 22)** | **Before** | **After** | **Before** | **After** |
| Age (years) | 46±10 | 57±12 | | 51±15 | |
| Sex (% female) | 77 | 78 | | 75 | |
| Disease duration (years) | NA | 8±6 | | 12±9 | |
| RF (+) % | ND | 78 | | 83 | |
| Anti-CCP (+) % | ND | 100 | | 92 | |
| CRP (mg/dl) | ND | 3.3±2.4 | 0.9±1.5 | 3.9±4.7 | 1.6±0.4 |
| ESR (mm/1^st^ hour) | ND | 53±24 | 20±9 | 49±18 | 7±5 |
| VAS | NA | 75±11 | 51±17 | 77±19 | 50±24 |
| DAS28 | NA | 5.0±1.3 | 4.1±1.8 | 4.9±1.8 | 3.0±1.2 |
| Swollen joints | NA | 6±4 | 2±3 | 5±4 | 2±2 |
| Tender joints | NA | 12±10 | 5±4 | 9±3 | 4±3 |

RF, Rheumatoid Factor; anti-CCP, anti-Cyclic Citrullinated Peptide Antibody; CRP, C-reactive protein; ESR, Erythrocyte Sedimentation Rate; VAS, Visual Analogue Scale; DAS28, Disease Activity Score of 28 joints; MTX, Methotrexate; NA, not applicable; ND, not determined.

**References**

[1] Shang, L.; Brandholt, S.; Stockmar, F.; Trouillet, V.; Bruns, M.; Nienhaus, G. U. Effect of protein adsorption on the fluorescence of ultrasmall gold nanoclusters. *Small* **8**:661-665; 2012.
